# Molecular and Clinical Characterization of a Novel Prognostic and Immunologic Biomarker GPSM3 in Low-Grade Gliomas

**DOI:** 10.3390/brainsci11111529

**Published:** 2021-11-18

**Authors:** Ming Wang, Jiaoying Jia, Yan Cui, Yong Peng, Yugang Jiang

**Affiliations:** Department of Neurosurgery, The Second Xiangya Hospital of Central South University, Changsha 410011, China; 188201059@csu.edu.cn (M.W.); jiajiaoying@csu.edu.cn (J.J.); 148202093@csu.edu.cn (Y.C.); foxpy195@csu.edu.cn (Y.P.)

**Keywords:** low-grade glioma, TME, immune cell infiltration, immunotherapy

## Abstract

**Background:** as the most common malignancy of the central nervous system, low-grade glioma (LGG) patients suffered a poor prognosis. Tumor microenvironment, especially immune components, plays an important role in the progression of tumors. Thus, it is critical to explore the key immune-related genes, a comprehensive understanding of the TME in LGG helps us find novel cancer biomarkers and therapeutic targets. **Methods:** the GPSM3 expression level and the correlations between clinical characteristics and GPSM3 levels were analyzed with the data from CGGA and TCGA dataset. Univariate and multivariate cox regression model were built to predict the prognosis of LGG patients with multiple factors. Then the correlation between GPSM3 with immune cell infiltration was explored by ESTIMATE, CIBERSORT and TIMER2.0. At last, the correlation analyzed between GPSM3 expression and immune checkpoint related genes were also analyzed. **Results:** GPSM3 expression was overexpressed in LGG and negatively correlated to the GPSM3 DNA methylation. Univariate and multivariate Cox analysis demonstrated that GPSM3 expression was an independent prognostic factor in LGG patients. Functional characterization of GPSM3 revealed that it was associated with many immune processes to tumor cells. GPSM3 expression was positive related to the immune score, Stromal scores and ESTIMATE scores, but negative related to the Tumor purity. Immune features in the TME of GPSM3-high LGG group is characterized by a higher infiltrating of regulatory T cells, neutrophils, macrophages M2, and a lower proportion of monocytes than to the GPSM3-low group. Furthermore, GPSM3 expression exhibited significant correlations with the immune checkpoint-related genes, especially PD-1, PD-L1, PD-L2, CTLA4 and TIM3. **Conclusions:** these findings proved that GPSM3 could serve as a prognostic biomarker and potential immunotherapy target for LGG.

## 1. Introduction

Glioma is the most common primary tumor of the central nervous system (CNS), accounting for slightly more than 50% of intracranial malignancies [1]. According to the World Health Organization (WHO) classification of tumors in the CNS, gliomas are classified into low-grade (LGG, WHO grades I–II) and high-grade (HGG, WHO grades III–IV) [2]. LGG is generally considered to be mainly composed of diffuse low-grade and intermediate-grade gliomas, including astrocytomas, oligoastrocytomas, and oligodendrogliomas [2]. At present, treatment of LGG usually includes traditional surgery, radiotherapy, and chemotherapy, but postoperative recurrence of tumor, transformation into GBM, and radiation-related complications result in a poor prognosis for patients with LGG.

The tumor microenvironment (TME) plays an important role in the progression, occurrence, and invasion of tumors [3]. Immune cells and stromal cells are the main non-tumor components of TME [3]. In the early stage of tumors, immune cells can be activated to inhibit tumor growth. As the tumor continues to stimulate and progress, immune and stromal components change, leading to immune tolerance and the formation of a microenvironment that promotes tumor progression [4,5]. Immunotherapy, a TME-targeted therapy, has emerged as a revolutionary treatment for cancers and has attracted increasing attention [6]. By targeting the immune checkpoint in TME, anti-cytotoxic T-lymphocyte associated protein 4 (CTLA4), anti-programmed cell death 1 (PD-1), and anti-CD274/PD-L1havebeen shown to be the most effective immunotherapy methods for cancer [7,8]. Unfortunately, only a small number of patients currently benefit from immunotherapy. Therefore, it is crucial to identify the more effective genes related to TME and the potential mechanisms of interaction between TME and glioma cells, which will help to improve the efficacy of immunotherapies for LGG.

Guanine-nucleotide-binding regulatory proteins (G-proteins) mainly relay the information from G-protein-coupled receptors (GPCRs) on the plasma membrane to the inside of cells to regulate numerous essential biological functions, especially in inflammatory responses and immune diseases [9]. The abnormal activation of G proteins is associated with the occurrence and development of diverse types of cancers [10]. The G protein signaling modulator family is the most important protein family that regulates the activation of G proteins, including GPSM1, GPSM2, and GPSM3 [11]. It has been reported that GPSM1 decreases the proliferation of esophageal squamous cell carcinoma and promotes tumor growth in B-cell acute lymphoblastic leukemia [12]. In addition, previous studies have reported the role of GPSM2 in several cancers, including hepatocellular carcinoma, pancreatic cancer, and breast cancer [13,14,15]. However, the role of GPSM3 in tumor progression and the underlying molecular mechanisms are poorly understood.

In this study, we first assessed the expression patterns and clinical characteristics of GPSM3 in LGG to determine its potential functions and prognostic values based on data from The Cancer Genome Atlas (TCGA) datasets and the Chinese Gliomas Genome Atlas (CGGA) datasets. Furthermore, the association between GPSM3 and immune cell infiltration was also explored, and we found that GPSM3 plays a role in various functional aspects of TME, especially its immune components, and may serve as a new potential bio-target for immune therapy.

## 2. Materials and Methods

### 2.1. Data Collection and Processing

We downloaded RNA-Seq gene expression profiles and clinical data of LGG samples from TCGA (https://www.cancer.gov/about-nci/organization/ccg/research/structural-genomics/tcga/using-tcga/citing-tcga, accessed on 10 November 2021) databases. The data were normalized and log2 transformed using package “DEseq.2” in R software. Gene expression data and corresponding clinical data of the mRNAseq_693datasetwere downloaded from the Chinese Glioma Genome Atlas (CGGA http://www.cgga.org.cn/, accessed on 10 November 2021) to verify our results based on data from TCGA. The TCGA dataset included 529 LGG samples and the CGGA dataset included 182 LGG samples. Samples with incomplete information and duplicates were removed.

### 2.2. Differential Expression Analysis and Survival Analysis

To investigate the role of GPSM3 in different cancers and the corresponding normal tissues, we analyzed the expression data of 33 human cancers in the TCGA dataset using the UCSCXenaShiny [16] (https://hiplot.com.cn/advance/ucsc-xena-shiny, accessed on 10 November 2021). Gene Expression Profiling Interactive Analysis (GEPIA, http://gepia.cancer-pku.cn/index.html, accessed on 10 November 2021) is a web application for analyzing gene expression in various tumor and normal samples from the Genotype Tissue Expression databases and the Cancer Genome Atlas databases [17]. We used GEPIA to investigate GPSM3 mRNA expression levels in normal and LGG tissues from the TCGA dataset. In addition, the ONCOMINE [18] gene expression array dataset (www.oncomine.org, accessed on 10 November 2021) was also downloaded to analyze the mRNA expression levels of GPSM3 mRNA in LGG and brain tissues. Furthermore, we analyzed the clinical features of different GPSM3 expression using CGGA mRNA expression and clinical datasets. The survival curve of GPSM3 expression was also analyzed by GEPIA to determine the correlation between GPSM3 expression and prognosis for patients with LGG, which was subsequently validated using the CGGA dataset. We also downloaded the methylation profiles of patients with LGG from the TCGA database via the Cbioportal website [19] (https://docs.cbioportal.org/1.-general/faq#how-do-i-cite-the-cbioportal, accessed on 10 November 2021) to observe the correlation between GPSM3 expression and GPSM3 DNA methylation.

### 2.3. Gene Set Enrichment Analysis (GSEA), Gene Set Variation Analysis (GSVA), and Functional Enrichment Analysis

In this study, GSEAsoftware20 (Version 4.1.0) [20] was used to study the biological processes associated with GPSM3.LGG samples from TCGA database and CGGA database were separately divided into low and high expression groups by setting the median of GPSM3 expression as a cut line. The R package “c2.cp.kegg.v7.0.symbols. gmt”was used as the reference gene set. For each analysis, 1000 repetitions of gene set permutations were performed. In this study, we found that KEGG signaling pathways were significantly enriched when the nominal P value was less than 0.01, |enrichment score (ES)| was greater than 0.5, gene size was50 or greater, and false discovery rate (FDR) was less than 25%. Gene Sets Variation Analysis (GSVA) [21] is a software used for gene set variation analysis, which can be downloaded from http://www.bioconductor.org, accessed on 10 November 2021. In order to determine which types of inflammatory activities were related to the expression of GPSM3, the relationship between seven metagenes [22] of 104 genes that were related to different types of inflammatory responses and GPSM3 were analyzed via GSVA in R. Metascape database [23] (http://metascape.org/gp/index.html#/main/step1, accessed on 10 November 2021) was also utilized to conduct functional enrichment analysis for the top 100 genes that positively correlated with the expression of GPSM3. Statistical significance was set at *p* < 0.05.

### 2.4. Immune Cell Infiltration Analysis

Estimation of Stromal and Immune cells in Malignant Tumors using Expression data (ESTIMATE) was used to evaluate the relationship between GPSM3 expression and tumor purity, as well as the presence of infiltrating stromal/immune cells. Tumor purity, stromal scores, ESTIMATE scores, and immune scores were calculated using the ESTIMATE algorithm for each sample. CIBERSORT (http://cibersort.stanford.edu/, accessed on 10 November 2021), a deconvolution algorithm based on gene expression can evaluate the changes in the expression of one set of genes relative to all other genes in the sample [24]. To assess whether infiltrating immune cells (TICs) in tumor samples were associated with the expression of GPSM3, we used CIBERSORT to estimate the relative abundance of 22 types of infiltrating immune cells. Tumor Immune Estimation Resource 2.0 (TIMER2.0) provides comprehensive analysis and visualization functions of tumor-infiltrating immune cells based on TCGA or user-provided tumor profiles [25] (https://cistrome.shinyapps.io/timer/, accessed on 10 November 2021). We investigated the association between GPSM3 expression in LGG and the abundance of immune cell infiltration, including B cells, CD4+ T cells, CD8+ T cells, neutrophils, macrophages, and dendritic cells via TIMER2.0.

### 2.5. Clinical Samples and RT-qPCR Analysis

In total, 10 LGG tumor tissues from glioma patients and five normal brain tissues from traumatic hematoma patients were obtained during neurosurgical procedures at the Second Xiangya Hospital of Central South University. The samples taken during the surgery were immediately frozen at −80 °C and then used for RNA isolation. The collection of human tissues was approved by the Ethical Committee of the Second Xiangya Hospital of Central South University, and written informed consent was obtained from all patients and their families. This study was conducted in accordance with the Declaration of Helsinki. Total RNA was extracted from LGG tumor and normal brain tissues using an RNA extraction kit (Bioteke Corporation, Beijing, China). For mRNA expression detection, the GoScript Reverse Transcription System (Promega Corporation, Madison, WI, USA) was used to reverse transcribe RNA templates, and the relative expression levels of mRNAs were determined with GoTaq qPCR Master mix (Promega Corporation, Madison, WI, USA) using a QuantStudio TM6 Flex real-time PCR system. The relative expression levels (fold change) were calculated using the 2^−ΔΔCq^ method, with GAPDH for normalization. The primers used were GAPDH F: 5′-GGTGGTCTCCTCTGACTTCAACA-3′ and R: 5′-GTTGCTGTAGCCAAATTCGTTGT-3′, GPSM3 F: 5′-AGTCACCAGTGCCAGCGGATG-3′ and R: 5′-GGGACCTTTGCTCCTCCATTCG-3′.

### 2.6. Statistical Analysis and Plot Generation

The R software (Version 4.0.3), BM SPSS Statistics 25 (Version 25.0.0.1), GraphPad Prism 8 software (Version 8.0.2), and Adobe Illustrator software (Version 24.0.2) were used to perform statistical analysis and generation of figures. Kaplan–Meier survival curves were drawn and compared among subgroups using log-rank tests with R packages “survival” and “survminer”. The Cox regression model was used for the univariate and multivariate analyses. ROC curves, sensitivity, and specificity were generated using the R package “pROC”. We calculated the correlations between the different variables using Spearman’s correlation test. Other R packages, namely “ggplot2”, “pheatmap”, “enrichplot”, and “corrplot”, were also applied for visualizing the results of data analysis. Statistical significance was set at *p* < 0.05.

## 3. Results

### 3.1. The mRNA Expression of GPSM3 in LGGs

**Figure 1:**
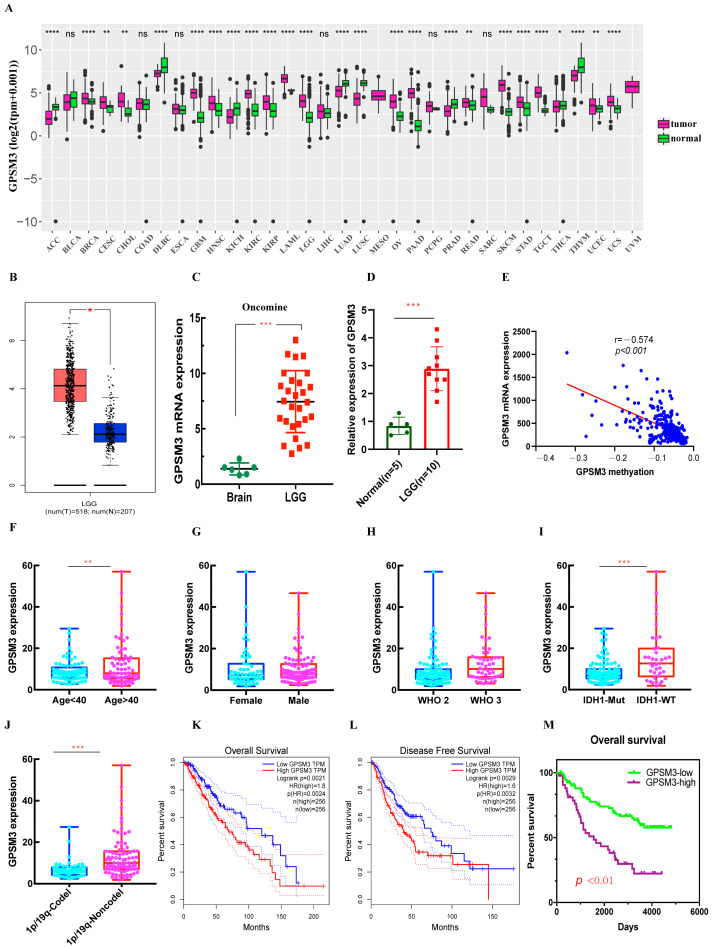
The mRNA Expression of GPSM3 in LGG and survival analysis. (**A**) The pan-cancer analyzed of GPSM3 mRNA expression, UCSCXenaShiny was used to visualize GPSM3 mRNA expression in the cancer genome atlas (TCGA) pan-cancer datasets. (**B**) The mRNA expression of GPSM3 in GEPIA website (Red color represent tumor tissues, Blue color represent normal tissues). (**C**) The mRNA expression of GPSM3 in dataset from ONCOMINE. (**D**) The mRNA expression of GPSM3 in LGG and normal brain tissues by PCR. (**E**) The expression of GPSM3 was negatively regulated by GPSM3 DNA methylation. (**F**–**J**) Correlation between GPSM3 mRNA expression and clinical indexes of LGG patients from CGGA database. ARL9 mRNA expression is stratified by age, gender, WHO grade, IDH1 mutation and 1p19q codeletion. * *p* < 0.05; ** *p* < 0.01; *** *p* < 0.001; **** *p* < 0.0001, ns = no significance. (**K**,**L**) Kaplan-Meier curves of low and high GPSM3 expression in LGG patients from TCGA dataset (Red color represent high GPSM3-LGG patients, Blue color represent low GPSM3-LGG patients). (**M**) Kaplan-Meier curves of low and high GPSM3 in LGG patients from CGGA dataset.

We first performed a pan-cancer analysis of GPSM3 in UCSCXenaShiny, and the results showed a significant difference in GPSM3 mRNA levels between a variety of tumors and normal tissues (Figure 1A). Expression of GPSM3 was higher in BRCA, GBM, HNSC, KIRC, KIRP, LAML, LGG, OV, PAAD, SKCM, STAD, TGCT, UCEC, and UCS (*p* < 0.05) than in normal tissues, whereas GPSM3 was lower in tumors of ACC, BLCA, DLBC, KICH, LUSC, PRAD, THCA, and THYM (*p* < 0.05). No difference in BLCA, COAD, ESCA, LIHC, PCPG, and SARC (*p* > 0.05) was observed when compared to normal adjacent tissues. In a subsequent study, we focused on exploring the expression and clinical value of GPSM3 in LGG. Based on the data from TCGA databases, the mRNA expression of GPSM3 in LGGs was significantly higher than that in normal brain tissues, which was further validated by the gene expression array data in the ONCOMINE database and our clinical specimens by PCR. (Figure 1B–D). To determine the underlying mechanism of GPSM3 overexpression in LGG, we further analyzed the relationship between DNA methylation and GPSM3 expression by Cbioportal. Finally, we observed a negative correlation (r = −0.574, *p* < 0.001) between GPSM3 expression and GPSM3 DNA methylation (Figure 1D), which indicates that GPSM3 overexpression in LGG may be due to some degree of inhibition of GPSM3 methylation. The clinical characteristics of GPSM3 in LGG were comprehensively analyzed using the CGGA dataset. The results showed a higher GPM3 mRNA expression in elderly patients than in young patients, but no significant difference in GPSM3 mRNA levels was indicated by sex and WHO grade (Figure 1E–G). It is well-known that isocitrate dehydrogenase (IDH) status and 1p19q codeletion influence the prognosis of gliomas; thus, we determined the expression patterns of GPSM3 based on IDH status and 1p19q codeletion. The results showed that the expression of GPSM3 was significantly upregulated in the IDH wild-type LGG and 1p19q codeletion LGG compared to that in IDH-mutant gliomas and 1p19q non-codeletion LGG (Figure 1H,I). Thus, the above results suggest that the expression of GPSM3 is positively correlated with the malignancy of LGG.

### 3.2. High Level of Expression of GPSM3 Predicted an Unfavorable Prognosis in the Patients with LGG

**Figure 2:**
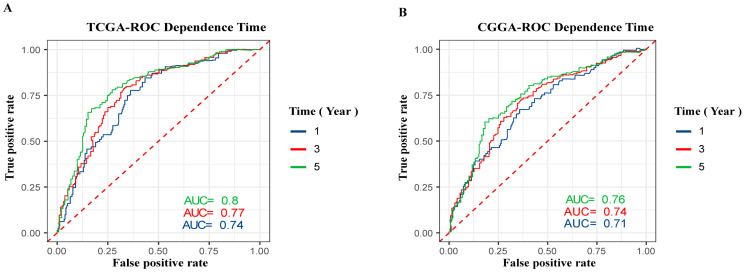
ROC curve analysis of GPSM3 in LGG patients. (**A**) ROC curve analysis of GPSM3 in LGG patients in the TCGA dataset. (**B**) ROC curve analysis of GPSM3 in LGG patients in the CGGA dataset. *p*-value < 0.05 was considered statistically significant.

To investigate the prognostic value of GPSM3 inpatients with LGG, we collected clinical and gene expression profile data from the TCGA and CGGA databases. The patients were divided into high and low expression groups based on the median value of GPSM3 mRNA expression. The subsequent Kaplan–Meier analysis indicated that the high GPSM3 expression group had shorter overall survival and disease-free survival in the patients with LGG from the TCGA cohort (*p* < 0.001, Figure 1G,K). In line with the results from the TCGA dataset, the patients with higher GPSM3 mRNA expression also exhibited shorter overall survival in the CGGA dataset (*p* < 0.001, Figure 1M). Additionally, the univariate and multivariate Cox analyses of the TCGA and CGGA cohorts indicated that age, IDH status, and GPSM3 expression could serve as independent prognostic factors in patients with LGG (*p* < 0.001, Table 1). Sex was an independent prognostic factor in the CGGA cohort (*p* < 0.001), while no prognostic value was observed in the TCGA cohort (*p* > 0.005). Moreover, we performed receiver operating characteristic (ROC) curve analysis to assess the predictive ability (1-, 3-, and 5-year overall survival) of GPSM3 in LGG. The areas under the ROC curve (AUC) for 1-year survival were 0.74 in the TCGA cohort and 0.71 in the CGGA cohort; 3-year survival were 0.77 in the TCGA cohort and 0.74 in the CGGA cohort; and 5-year survival were 0.8 in the TCGA cohort and 0.76 in the CGGA cohort (Figure 2A,B). Overall, the above results suggest that GPSM3 can be an important prognostic biomarker for patients with LGG.

### 3.3. The Potential Functions of GPSM3

**Figure 3:**
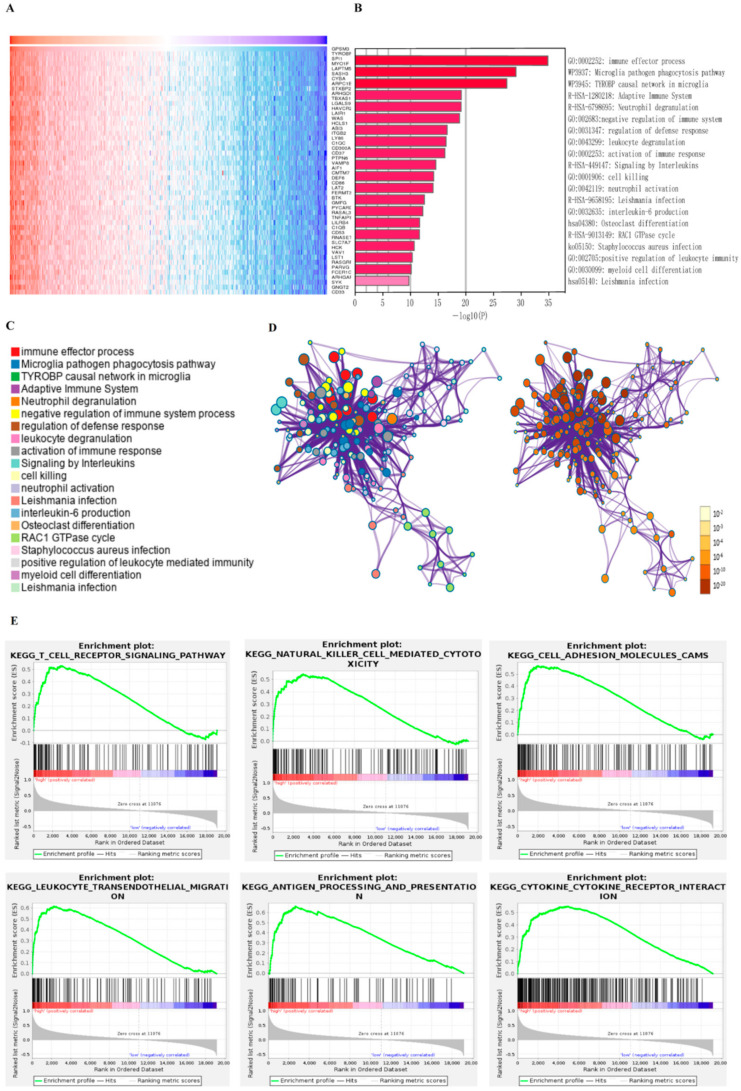
The function analysis of GPSM3 in LGG patients. (**A**) Heatmap of GPSM3 top 50 positively correlated genes. (**B**) Bar graph showing the enriched terms of GPSM3 positively correlated genes. The color depth denoted the *p*-value. (**C**,**D**) Network plot of enriched terms: (**C**) each node represented one enriched term colored by its cluster ID; (**D**) colored by *p*-value. (**E**) GSEA enrichment analysis of GPSM3 high expression group in TCGA dataset, *p*-value < 0.05 was considered statistically significant.

To explore the potential functions of GPSM3, we examined the correlation between GPSM3 and other genes in the LGG gene expression profile using the online database LinkedOmics (Figure 3A). The top 100 positively correlated genes were selected for enrichment analysis using Metascape online tools. As presented in Figure 3B–D, these genes were primarily enriched in immune effector process, adaptive immune system, negative regulation of immune system, activation of immune response, and leukocyte degranulation, some of which exhibited immunologic characteristics. GSEA was performed to distinguish the signaling pathways involved in the LGGs between the high and low GPSM3 expression groups. As shown in Figure 3E, the T cell receptor signaling pathway, natural killer cell-mediated cytotoxicity, cell adhesion molecules, leukocyte transendothelial migration, antigen processing and presentation, and cytokine-cytokine receptor interaction were enriched in the GPSM3 high expression group from the TCGA cohort. Similar results were obtained in the CGGA dataset. The top 10 KEGG signaling pathways in these two cohorts are listed in Table 2. Based on our findings, GPSM3 appears to serve as a crucial factor in the regulation of immune-related biological processes in the LGG microenvironment.

### 3.4. GPSM3 Regulated the Infiltration of Immune Cells in the LGGs

**Figure 4:**
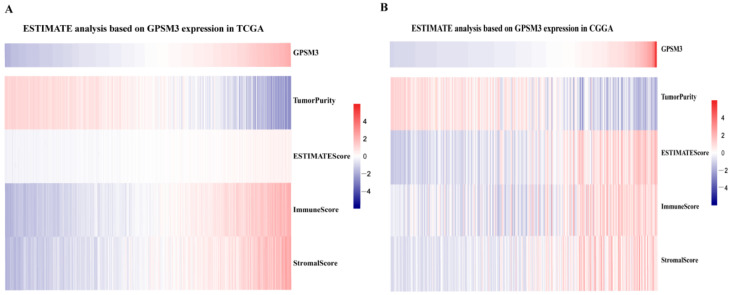
The ESTIMATE analysis of GPSM3 in LGG patients. (**A**) GPSM3 was positively related to the Stromal scores and ESTIMATE scores, but negative related to the Tumor purity in the TCGA dataset. (**B**) GPSM3 was positively related to the Stromal scores and ESTIMATE scores, but negative related to the Tumor purity in the CGGGA dataset.

**Figure 5:**
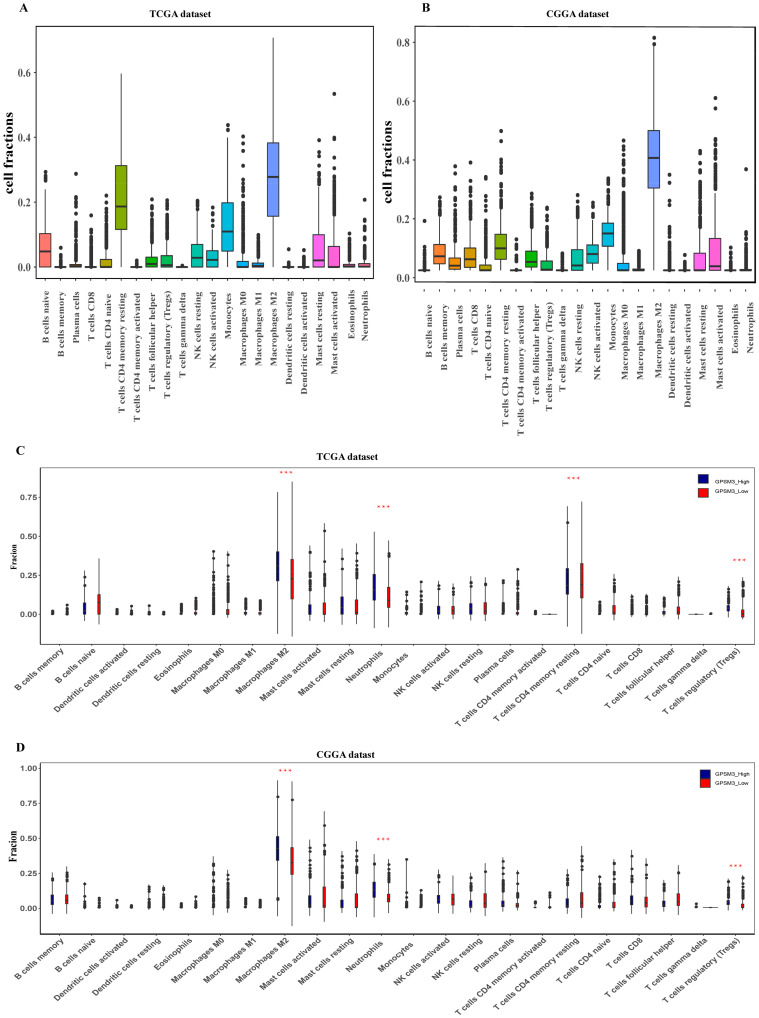
GPSM3-related immune population in the TME of LGG. (**A**,**B**) Bar plots showing the proportion of 22 immunocyte types in TCGA dataset and CGGA dataset. (**C**,**D**) Violin plots showing the differences in the proportion of 22 immunocyte types between GPSM3 high and low expression groups. Blue colors represented the group with GPSM3 high expression; Red color represented the group with GPSM3 low expression. The symbol *** indicated *p* < 0.001.

**Figure 6:**
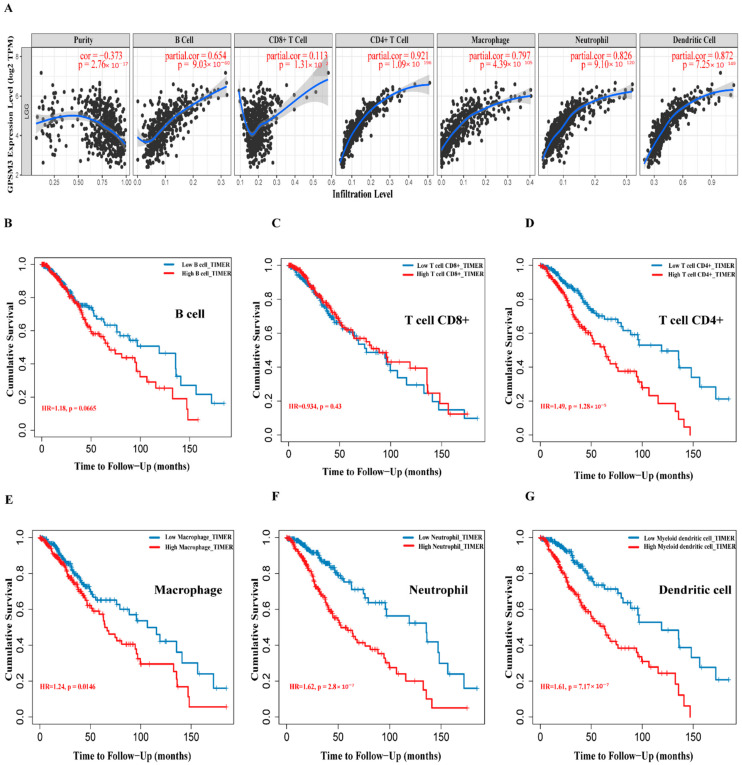
Correlation between GPSM3 expression and immune cell infiltration. (**A**) The correlation between the GPSM3 expression level and infiltrating levels of B cell (r = −0.654, *p* = 9.03 × 10^−6^), CD8+ T cells (r = −0.113, *p* = 1.31 × 10^−2^), CD4+ T cells (r = −0.921, *p* = 1.09 × 10^−196^), Macrophages (r = −0.797, *p* = 4.39 × 10^−105^), Neutrophils (r = −0.862, *p* = 9.10 × 10^−120^), and DCs (r = −0.872, *p* = 7.25 × 10^−149^) in LGG. (**B**–**G**) Cumulative survival analysis related to the infiltration of B cell, T cells, Macrophages, Neutrophils and DCs in LGG. *p*-Value < 0.05 was considered statistically significant.

Considering that GPSM3 might play a crucial role in regulating immune-related biological processes in the LGGs, the ESTIMATE method was used to investigate the relationship between GPSM3 expression and immune cell infiltration in the TCGA and CGGA datasets. The results showed that GPSM3 expression was positively related to the immune score, stromal scores, and ESTIMATE scores in the TCGA dataset, but negatively correlated with tumor purity (Figure 4A). Similar results were confirmed in the CGGA database (Figure 4B), which indicated that the expression of GPSM3 plays a regulatory role in the TME of LGG. Therefore, we further examined the abundance of 22 types of infiltrating immune cells in the TCGA and CGGA datasets using the CIBERSORT algorithm. The results showed that M2 macrophages accounted for the largest proportion of the 22 immune cell types, suggesting the involvement of these cell types in the development of LGG (Figure 5A,B). In addition, the proportion of 22 immune cell profiles in the high GPSM3 expression and low GPSM3expression groups were also compared. Notably, the proportions of immune cell profiles showed marked variations between the GPSM3 high and low subgroups. As shown in Figure 5C, M2 macrophages, neutrophils, monocytes, and regulatory T cells (Tregs) were the main immune cells affected by GPSM3 expression in the TCGA dataset. Among them, M2 macrophages, neutrophils, and Tregs were apparently increased, but monocytes were decreased in the GPSM3-high group compared with the GPSM3-low group; similar results were obtained in the CGGA dataset (Figure 5D), changes in the proportion of these immune cells may contribute to the immunosuppressive microenvironment of LGG. Finally, the correlation between immune cell infiltration and GPSM3 expression was estimated using TIMER. As illustrated in the scatter plots (Figure 6A), the expression of GPSM3 was positively correlated with immune infiltration of CD4+ T cells, B cells, dendritic cells, macrophages, and neutrophils. Moreover, Kaplan–Meier survival analysis demonstrated that patients with LGG with low immune infiltration of CD4+ T cells, macrophages, neutrophils, and dendritic cells exhibited appreciably longer overall survival than those with high levels (Figure 6B–G). These results further support the hypothesis that the expression of GPSM3 affects the immune response of the TME in patients with LGG.

### 3.5. GPSM3-Related Inflammatory Responses

**Figure 7:**
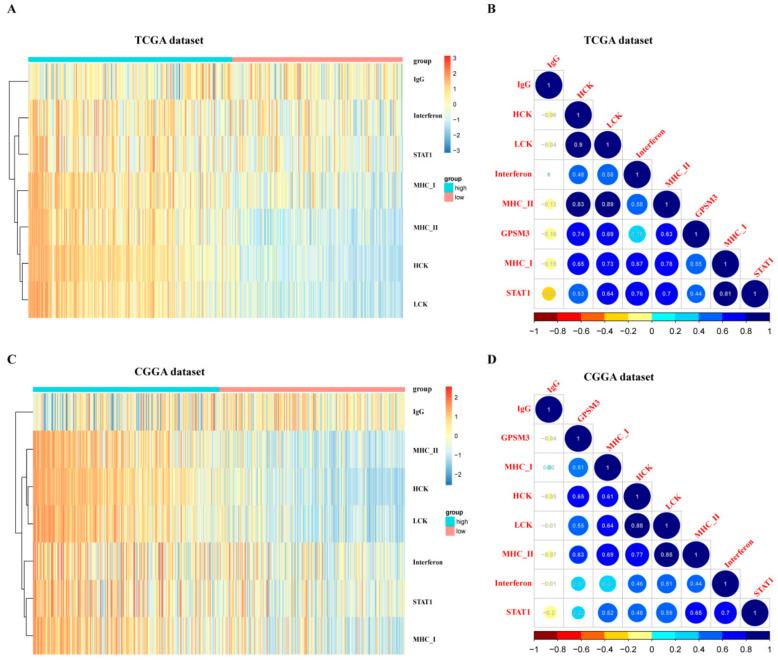
GPSM3-related inflammatory activities in LGG. (**A**,**B**) Heatmaps showing the relationship between GPSM3 and seven metagenes in TCGA and CGGA datasets. (**C**,**D**) Correlogram showing the correlation between GPSM3 and seven metagenes in TCGA and CGGA datasets.

Inflammation also plays an important role in the host immune response to tumors, as well as in cancer immunotherapy. To determine which types of inflammatory activities were related to GPSM3, we further analyzed the association between GPSM3 and different inflammatory responses. Therefore, seven clusters of metagenes representing different types of inflammatory and immune responses were selected to analyze the association between GPSM3 and different inflammatory responses. GSVA was subsequently performed to convert the expression data of these metagenes into enrichment scores. As shown in the heatmap, GPSM3 was positively associated with HCK, LCK, MHC I, and MHC II, both in the CGGA and TCGA datasets (Figure 7A,C).The correlogram was used to display the correlation between the seven inflammatory metagene signatures and GPSM3 (Figure 7B,D). This analysis showed that GPSM3 was positively related to the signature of HCK (r = −0.74/0.65), LCK (r = −0.69/0.55), MHCI (r = −0.63/0.51), and MHCII (r = −0.55/0.63).Overall, based on our results, GPSM3 appears to be closely associated with inflammatory responses in LGGs.

### 3.6. Correlation Analysis between GPSM3 and Immune Checkpoints

**Figure 8:**
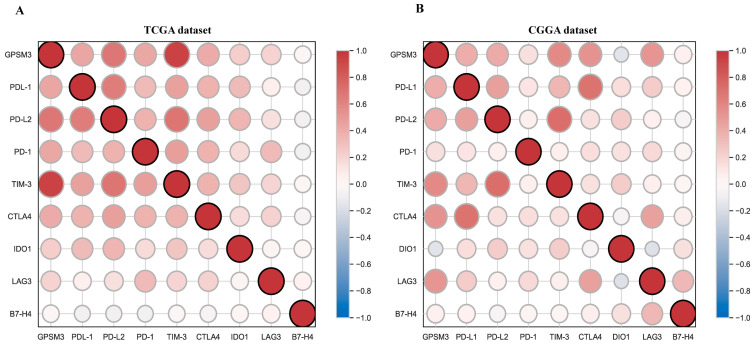
Correlation analysis between GPSM3 and immune checkpoints. (**A**) Correlation matrix plots of GPSM3 and major immune checkpoints in the TCGA dataset. (**B**) Correlation matrix plots of GPSM3 and major immune checkpoints in the CGGA dataset.

Immune checkpoints play a crucial role in tumor immunosuppression. Therefore, we analyzed the relationship between expression of GPSM3 and immune checkpoint-related genes by Pearson correlation analysis in LGG, including PD-1, PD-L1, PD-L2, TIM-3, CTLA4, IDO1, LAG3, and B7-H3. Correlation matrix plots indicated that GPSM3 was significantly correlated with several immune checkpoints in the TCGA database. Notably, GPSM3 showed significant positive relationships with PD-1, PD-L1, PD-L2, TIM3, and CTLA4 in TCGA datasets (Figure 8A), and similar results were also obtained in the CGGA datasets (Figure 8B).In short, these results suggest that GPSM3 may be a good index for quantifying the TME, especially its immune components, and predicting the immunotherapy responses of LGG.

## 4. Discussion

Although it is the most common malignancy of the CNS, most patients with LGG have a poor prognosis [26]. In recent years, immunotherapy targeting the immune checkpoints, also called immune checkpoint blockade (ICB), has shown effectiveness against a variety of cancers [6]. However, some malignant tumors characterized by systemic immunosuppression, such as gliomas, often fail to achieve good results from ICB [27]. The TME, especially its immune components, plays an important role in the progression of tumors [3]. Thus, it is critical to explore the key immune-related genes. A comprehensive understanding of the TME related to the target genes in LGG will help improve the efficacy of immunotherapies in LGG. In the present study, we discovered that GPSM3 expression can predict the prognosis for patients with LGG and is closely related to the regulation of the immune microenvironment.

The Gprotein signaling pathway is involved in tumorigenesis and development [13]. The G protein signaling modulator family is the most important protein family that regulates the activation of G proteins, including GPSM1, GPSM2, and GPSM3 [11]. Previous studies have reported the role of GPSM1 and GPSM2 in several cancers, including hepatocellular carcinoma, pancreatic cancer, and breast cancer [12,13,14,15]. However, few studies have clarified the role of GPSM3 in LGG. In the present study, we first performed a pan-cancer analysis in UCSCXenaShiny to explore the role of GPSM3 in cancers and observed that GPSM3is highly expressed in a variety of cancers, which may indicate its crucial role in cancers. Subsequently, we confirmed the higher expression of GPSM3 in LGG using two different tumor databases, GEPIA and ONCOMINE. Increasing evidence has shown that abnormal DNA methylation plays an essential role in the induction and progression of LGG [28]. In the present study, we found that GPSM3 expression negatively correlated with GPSM3 DNA methylation. In addition, in the multivariate Cox analysis, both the expression of GPSM3 and GPSM3 methylation was found to be a prognostic factor for the overall survival in LGG. Our results indicate that GPSM3 DNA methylation may be the key pathway to regulating the expression of GPSM3, activation of GPSM3 DNA methylation may contribute to the improvement of immunotherapy efficacy for LGG, but this needs to be further confirmed in subsequent studies. IDH wild-type gliomas are often associated with poor prognosis [29]. Subgroup analyses were further implemented based on patient sex, age, WHO grade, IDH1-mutation, and 1p19q-Codelstatus. We observed that higher ARL9 expression was more closely correlated with the IDH1-mutation status and 1p19q-Codelstatus. Cox regression models also indicated the critical role of IDH1-mutation status in the unfavorable prognosis for patients with LGG. Moreover, we also verified the prognostic value of GPSM3 expression in CGGA and TCGA datasets by ROC analysis, and the results revealed that GPSM3 could function as a sensitive indicator for predicting 1-year, 3-year, and 5-year survival rates for patients with LGG. These results emphasize the promising prognostic value of GPSM3 expression in patients with LGG.

To further investigate the potential mechanisms of GPSM3 in LGGs, we enriched the pathways of the top 100 genes that were positively related to GPSM3 in the LGG via LinkedOmics. The results indicated that genes related to GPSM3 were the most involved in the immune process. GSEA was performed to investigate the potential functions of GPSM3. Likewise, the GSEA results demonstrated that many immune response-related processes were enriched in the GPSM3 higher group. Thus, GPSM3 is an immune-related gene and is involved in the immune processes in the TME of LGGs. Immune cells regulate the TME and affect tumor growth and prognosis. In this study, the relationship between the expression of GPSM3 and immune features in the TME of LGG was explored. Glioma tissues contain abundant glioma-associated non-tumor cells within their microenvironment, the purity of glioma cells can be diluted by non-tumor cells, these non-tumor cells play important roles in glioma development and are represented by stromal and immune cells [30]. We first used the ESTIMATE method to investigate the relationship between GPSM3 expression and immune cell infiltration in the TCGA and CGGA datasets. The results showed that GPSM3 expression was positively related to the immune score, stromal scores, and ESTIMATE scores, but negatively related to tumor purity, indicating that the expression of GPSM3 plays a regulatory role in the TME component in LGG. We further examined the abundance of 22 types of infiltrating immune cells in the TCGA and CGGA datasets using the CIBERSORT algorithm and the TIMER algorithm. In the CIBERSORT analysis results, we observed that the GPSM3-high group was characterized by a higher proportion of regulatory T cells, neutrophils, and M2 macrophages, and a lower proportion of monocytes than the GPSM3-low group, and the M2 macrophages accounted for the largest proportion of the 22 immune cell types in the TME of LGG. In addition, our TIMER analysis revealed that expression of GPSM3 was positively correlated with most of the immune cells, except for CD8+ T cells, and that a higher infiltration level of CD4+ T cells, neutrophils, macrophages, and dendritic cells was correlated with a poor survival rate. Immune cells in the TME can be divided into those that promote tumor growth and those inhibiting tumor growth [31]. Macrophages have been demonstrated to play an important role in tumor growth and angiogenesis [32]. Macrophages can produce immunosuppressive cytokines to induce the formation of M2 macrophage-like functional status, which modulates adaptive immunity and promotes angiogenesis, and thus, affect tumor proliferation and metastasis [33]. Studies have also shown that neutrophils play different roles in cancer development and progression [34]. For example, it has been reported that neutrophils promote the malignant glioma phenotype through S100A4 [35]. The role of regulatory T cells (Tregs) in mediating immune suppression of anti-tumor immune responses is increasingly appreciated in patients with malignancies, especially within the malignant glioma patient population [36]. For example, research has revealed that Tregs express Foxp3 to inhibit the response of immune cells to tumors [37]. It is interesting that all three immune cells were highly infiltrated in LGG with high GPSM3 expression, which may contribute to the immunosuppressive microenvironment of LGG. Achim Rody et al. identified seven metagenes associated with immuneresponse in tumors [22]. In the analysis of GPSM3 and seven immune metagenes, we found that GPSM3 expression was particularly correlated with macrophage-related LCK and T cell-related HCK and MHC II, but not with B cell-related IgG. These results suggest that GPSM3plays an important role in the regulation of immune responses. In summary, our immune cell infiltration study implied the role of GPSM3 in remodeling the immune microenvironment of LGG.

ICB uses immune checkpoint inhibitors to block inhibitory signaling and directly stimulate the activation of cytotoxic T lymphocytes to achieve anti-tumor effects [38]. Given the importance of immunotherapy in cancers, we analyzed the correlation between GPSM3 and immune checkpoint genes in the TCGA-LGG and CGGA-LGG datasets. Indeed, GPSM3 exhibited significant correlations with immune checkpoints, especially PD-1, PD-L1, PD-L2, CTLA4, and TIM3. It has been demonstrated that CTLA4, PD1, and PDL1 as immune checkpoints can prevent the immune system from killing cancer cells by inhibiting auto-immunity [7,8]. Therefore, the use of anti-CTLA4, anti-PD1, and anti-PDL1 drugs has become the most effective method for ICB therapy by regulating the state of T cells. Our results suggest that GPSM3 is closely linked to an immunosuppressive phenotype and may be a potential predictive marker for ICB response.

## 5. Conclusions

GPSM3 is highly expressed in LGG and is correlated with an unfavorable prognosis. The level of GPSM3wasassociated with the immune response and an immunosuppressive TME in LGG. These novel findings indicate that it is a potential target for immunotherapy in patients with LGG.

## Figures and Tables

**Table 1:** Univariate and multivariate Cox analysis of clinic-pathologic characteristics in LGG based on CGGA and TCGA datasets.

Datasets	Characteristic	HR	95% CI	*p*-Value		HR	95% CI	*p*-Value
**TCGA**	**Univariate**				**Multivariate**			
	Age	2.89	1.98–4.23	0.000	Age	3.24	2.14–4.91	0.000
	Ethnicity	1.26	0.46–3.44	0.645	Ethnicity	0.92	0.32–2.57	0.873
	Gender	1.07	0.75–1.53	0.692	Gender	1.19	0.83–1.74	0.336
	Radiation	2.07	0.89–4.79	0.088	Radiation	1.05	0.72–1.53	0.786
	GPSM3	2.41	1.65–3.54	0.000	GPSM3	2.08	1.36–3.21	0.0001
**CGGA**	**Univariate**				**Multivariate**			
	Gender	2.91	1.92–4.41	0.000	Gender	1.61	1.07–2.41	0.019
	Age	1.27	0.90–1.81	0.016	Age	1.44	0.99–2.09	0.031
	Radio_status	1.37	0.89–2.09	0.145	Radio_status	1.27	0.78–2.06	0.328
	Chemo_status	0.94	0.64–1.38	0.788	Chemo_status	0.64	0.41–1.00	0.448
	IDH_mutation	2.18	1.50–3.16	0.000	IDH_mutation	2.34	1.50–3.66	0.000
	X1p19q_codeletion	2.78	1.78–4.33	0.000	X1p19q_codeletion	1.08	0.51–2.28	0.831
	MGMTp_methylation	1.16	0.82–1.64	0.038	MGMTp_methylation	0.91	0.62–1.34	0.041
	GPSM3	1.24	0.878–1.76	0.000	GPSM3	1.09	0.73–1.63	0.005

**Table 2:** Genet set enrichment analysis (GSEA) in GPSM3 high-expression LGG.

TCGA-KEGG	SIZE	ES	NES	NOM *p*-Value	CGGA-KEGG	**SIZE**	**ES**	**NES**	**NOM *p*-Value**
LEUKOCYTE_TRANSENDOTHELIAL_MIGRATION	116	0.61	2.19	0	ANTIGEN_PROCESSING_AND_PRESENTATION	80	0.68	2.38	0
B_CELL_RECEPTOR_SIGNALING_PATHWAY	75	0.61	2.05	0	PRIMARY_IMMUNODEFICIENCY	65	0.77	2.36	0
PRIMARY_IMMUNODEFICIENCY	65	0.74	2.01	0	INTESTINAL_IMMUNE_NETWORK_FOR_IGA	56	0.62	2.29	0
ACUTE_MYELOID_LEUKEMIA	57	0.52	1.77	0.001	CYTOKINE_RECEPTOR_INTERACTION	115	0.63	2.26	0
CELL_ADHESION_MOLECULES_CAMS	131	0.56	1.93	0.001	B_CELL_RECEPTOR_SIGNALING_PATHWAY	74	0.61	2.22	0
NATURAL_KILLER_CELL_MEDIATED_CYTOTOXICITY	132	0.54	1.96	0.004	TOLL_LIKE_RECEPTOR_SIGNALING_PATHWAY	101	0.58	2.19	0.001
ANTIGEN_PROCESSING_AND_PRESENTATION	81	0.66	2.02	0.004	CELL_ADHESION_MOLECULES_CAMS	128	0.55	2.06	0.004
T_CELL_RECEPTOR_SIGNALING_PATHWAY	107	0.52	1.84	0.005	NATURAL_KILLER_CELL_MEDIATED_CYTOTOXICITY	131	0.55	2.04	0.004
CYTOKINE_CYTOKINE_RECEPTOR_INTERACTION	263	0.55	1.92	0.006	JAK_STAT_SIGNALING_PATHWAY	151	0.55	2.04	0.005
NOD_LIKE_RECEPTOR_SIGNALING_PATHWAY	62	0.56	1.84	0.009	T_CELL_RECEPTOR_SIGNALING_PATHWAY	105	0.54	1.99	0.006

## Data Availability

Publicly available datasets were used in this study. The datasets analyzed in the present study were retrieved from The Cancer Genome Atlas and Chinese Glioma Genome Atlas databases.
